# Resistance Inducers Modulate *Pseudomonas syringae* pv. Tomato Strain DC3000 Response in Tomato Plants

**DOI:** 10.1371/journal.pone.0106429

**Published:** 2014-09-22

**Authors:** Loredana Scalschi, Gemma Camañes, Eugenio Llorens, Emma Fernández-Crespo, María M. López, Pilar García-Agustín, Begonya Vicedo

**Affiliations:** 1 Grup de Bioquímica i Biotecnologia, Area de Fisiologia Vegetal, Departament de Ciències Agràries i del Medi Natural, ESTCE, Universitat Jaume I, Castellón, Spain; 2 Departamento de Protección Vegetal y Biotecnología, Instituto Valenciano de Investigaciones Agrarias (IVIA), Apartado Oficial, Moncada, Valencia, Spain; Niels Bohr Institute, Denmark

## Abstract

The efficacy of hexanoic acid (Hx) as an inducer of resistance in tomato plants against *Pseudomonas syringae* pv. tomato DC3000 was previously demonstrated, and the plant response was characterized. Because little is known about the reaction of the pathogen to this effect, the goal of the present work was to determine whether the changes in the plant defence system affect the pathogen behaviour. This work provides the first demonstration of the response of the pathogen to the changes observed in plants after Hx application in terms of not only the population size but also the transcriptional levels of genes involved in quorum sensing establishment and pathogenesis. Therefore, it is possible that Hx treatment attenuates the virulence and survival of bacteria by preventing or diminishing the appearance of symptoms and controlling the growth of the bacteria in the mesophyll. It is interesting to note that the gene transcriptional changes in the bacteria from the treated plants occur at the same time as the changes in the plants. Hx is able to alter bacteria pathogenesis and survival only when it is applied as a resistance inducer because the changes that it promotes in plants affect the bacteria.

## Introduction

Resistance-inducing agents that activate the natural defences of plants have been identified, and it is known that their application protects plants through the induction of defences that are effective against a wide spectrum of pathogens. Their mechanism of action has been studied in different pathosystems over the last years [1]–[6]. However, all of the previous studies were focused on plant defence responses, and little is known about how the pathogen reacts against the induction of plant defences.

Previous investigations by our research group have shown that the resistance-inducer hexanoic acid (Hx) protects *Solanum lycopersicum* plants against the necrotroph *Botrytis cinerea*, a pathogen that has proven difficult to chemically control [1], [6]. Furthermore, its efficacy was demonstrated in *Arabidopsis thaliana* [7]. We also observed an approximately 50% reduction in disease symptoms against *Pseudomonas syringae* pv. tomato strain DC3000 (*Pst* DC3000) in treated plants compared with non-treated plants [6], which suggests that Hx induces resistance against this pathogen. The mechanisms involved in the induction of resistance have been studied in plants [4], but the bacterial response to this process remains unelucidated. Therefore, the present study was undertaken to gain insight into the mechanism of action of Hx, particularly its effect on bacterial pathogens.

We chose the plant pathogen *Pst* DC3000 because it represents an important pathogen that infects economically important crops. In nature, this pathogen causes bacterial speck disease in tomato and bell pepper and can also infect *Arabidopsis* and *Brassica* species in the laboratory [8], [9]. Moreover, *Pst* DC3000 is considered a model organism for molecular studies of plant-pathogen interactions [10], [11].

The infection cycle of *Pst* DC3000 is typical for a foliar pathogenic bacterium. In a successful disease cycle, *Pst* DC3000 generally shows two lifestyles that are spatially and temporally interconnected: an initial epiphytic phase and a subsequent endophytic phase in the apoplast [12]. Before initiating disease, *Pst* DC3000 strains achieve large epiphytic population sizes on healthy leaves [13]. Bacterial populations are capable of coordinating the expression of some traits in a cell-density-dependent manner through the process known as quorum-sensing (QS) [14], [15]. *Pst* DC3000 and many other Gram-negative bacteria use N-acyl homoserine lactone (AHL) as the QS signal molecule [16]. In addition, *psyI* of *Pst* DC3000 functions as an AHL synthase, and *psyI* expression, as well as AHL production, are under the control of the sigma regulator PsrA. Elevated levels of AHL due to higher population densities result in the expression of virulence factors and secondary metabolites that mediate colonization of the host.

*Pst* DC3000 contains more than thirty type III effectors [17], [18]. One of the most important effectors for the virulence of the bacteria is AvrPtoB (hopAB2), which has been found to act inside plant cells to suppress programmed cell death associated with plant immunity and increases pathogen growth and disease formation [19]. The effectors, together with the T3SS apparatus, are encoded by the hrp/hrc regulon. The *hrpL* gene encodes the alternate RNA polymerase sigma factor σL, which is one of the primary transcription factors that modulate the expression of the T3SS [20].

*Pst* DC3000 produces a polyketide toxin named coronatine (COR) [21]. COR is a chlorosis-inducing phytotoxin that contributes to bacterial growth and lesion formation or expansion in the host tissue. COR acts as a virulence factor in various *P. syringae* pathovars by generating increased lesion size and bacterial multiplication [22]. Several studies have shown that COR plays a critical role in pathogenesis [23], [24]. Moreover, it is known that COR controls stomata opening upon bacterial infection [12]. COR consists of two distinct moieties that are synthesized by separate pathways, and an amide linkage then joins the two moieties: the polyketide coronafacic acid (CFA) and coronamic acid (CMA), an ethylcyclopropyl amino acid derived from isoleucine [25].

The role of CorA, the major magnesium transporter, in pathogenesis has been studied in a number of organisms, such as *Salmonella enterica* serovar *typhimurium*, in which it is required for full virulence [26]. A role for CorA has also been established in the human gastric pathogen *Helicobacter pylori*, where it is required for viability under limiting Mg^2+^ conditions [27]. In addition to being necessary for bacterial growth, magnesium is a cofactor in ATP-requiring enzymatic reactions [28] and is directly involved in membrane stability [29].

In this study, we investigated whether the resistance inducer Hx displays any direct antimicrobial effect on the target bacterium by performing *in vitro* assays with different Hx concentrations to determine its effect on bacterial growth, viability, and gene expression. Moreover, the effect of Hx treatment on the bacteria during plant infection was also investigated through an analysis of the expression of genes related to the different components of pathogenicity and virulence explained above. From our *in vitro* experiments, we can conclude that higher concentrations of Hx may have a temporary bacteriostatic effect against *Pst* DC3000 or a short-term bactericidal effect.

On the other hand, we previously observed that Hx treatment against *Pst* DC3000 affects the JA and SA pathways and prevents stomata opening induced by coronatine *in planta* [4]. Concurrently, in this study, we noticed a reduction in the expression of bacterial genes involved in quorum sensing establishment and pathogenesis, such as COR synthesis genes, genes associated with the type III secretion system, and genes responsible for effector synthesis in treated plants.

## Materials and Methods

### Bacterial strains and growth conditions

The *P. syringae* pv. tomato strain used in the present study was DC3000, rifampicin resistent. Rifampicin was added to King B medium (KB) at 50 µg mL^−1^. Mutants used were Δ*hrpL*, (Beuzon, C. Dep. Biología Celular, Genética y Fisiología Universidad de Malaga, Spain), Δ*hrpA* (He, S. Dep. of Plant Pathology, University of Kentucky, Lexington), Δ*cma-cfa* (Brooks, D. Department of Biology, Washington University, St. Louis) and Δ*pto(ΔavrPto*-*ΔavrPtoB*) (Martin, G. Boyce Thompson Institute for Plant Research, Tower Rd., Ithaca,). They were grown in King's B medium (KB) [54] at 28°C with their specific antibiotics.

### Bacterial growth assay

We tested Hx effect against *Pst* DC3000 and several mutants. Long time experiments were performed in LB medium to which, the resistance inducer was added at a final concentration of 0.6, 1.5, 5, 10 and 20 mM. pH of medium was adjusted to 7 after Hx was added and before adding the bacteria. The strain was precultivated in LB overnight to obtain the inoculum. The bacteria were harvested by centrifugation, washed and resuspended in sterilized MgSO_4_ (10 mM).

The growth assay was carried in a Bioscreen C analysator (Labsystem, Finland). In a total volume of 350 µl in microtiter wells using an initial bacterial density of about 1×10^3^ cfu/mL. Bacterial growth was monitored by measuring optical density every 10 min with periodic shaking for 96 h at 26°C. The results were printed out as growth curves.

Moreover Hx effect to short time points was studied with M9 medium (Sigma) to wich Hx was added at a final concentration of 10 and 20 mM. Bacteria were preincubated as described above and added to final concentration of 1×10^3^ cfu/ml. *Pst* DC3000 was incubated with shaking for 16 h to 26°C. After this time, live and dead cell were quantified as described below.

### Live and dead cell quantification

The proportion of living vs. dead cells was quantified using the fluorescent LIVE/DEAD BacLight Bacterial Viability Kit, L13152 (Molecular Probes, Invitrogen, Paisley, UK). For live and dead cell quantification, 50 µl of bacterial suspension were mixed with 25 µl of each of the two components of the LIVE/DEAD BacLight kit and incubated in the dark for 20 min. The viability of the cells was determined by enumerating the live and dead cells using an epifluorescence microscope Leica IRB equipped with a Leica DBC300F camera (Leica Microsystems CMS GmbH, Wetzlar, Germany). Live and dead cells were enumerated in 15 microscopic fields at 1000× magnification.Two independent experiments were performed with five repetitions for each treatment.

### Plant inoculation

Four-week-old tomato plants of *Solanum lycopersicum* L. cv. Ailsa Craig were treated with Hx and inoculated as described by Scalschi *et al*. [4].Hx treatment was applied by soil drench to 4-week-old plants grown in jiffy peat pellets at a concentration of 0.6 mM, 72 h before bacterial inoculation. For inoculation, Pst DC3000 was grown in KB at 28°C for 24 h. Bacterial suspensions were adjusted to 5×10^5^colony-forming units (CFU)/mL in sterile MgSO_4_ (10 mM) containing 0.01% of the surfactant Silwet L-77 (Osi Specialties, Danbury, CT, USA), as described previously [11].Pathogen inoculation was performed by dipping the third and fourth leaves into the bacterial suspension.

The disease rate was scored at 72 hpi by determining the percentage of dark-brown spots on the leaf surface and by counting the colony forming units in KB medium. At least three samples for colony counting and 20 samples for disease rate scoring were taken for each treatment over a 3-day period. For bacteria gene expression analysis, samples were taken at 2, 6, 10, 18, 48 and 72 h after inoculation. Each experiment was independently conducted at least three times.

Long term experiments were performed in the same conditions like those described above for 30 days. The samples were taken at 4, 10, 20 and 30 days after inoculation. Each experiment was independently conducted at least three times.

### Bacterial RNA extraction and cDNA synthesis

Bacterial RNA extractions were carried out from *in vitro*-cultured cells after 18 h and from cells grown *in planta* at different time intervals.

For RNA isolation from *in vitro* cultured bacteria, *Pst* DC3000 was grown on KB agar plates for 48 h at 28°C. Hx was added to 1×10^6^ bacterial cells suspensions to final concentrations of 1.5 mM, 5 mM and 10 mM. Cells were incubated by shaking for 18 h before RNA extraction. Bacterial suspensions were stabilized with RNA protect bacterial reagent (Qiagen) before centrifugation. Cells pellets were immediately frozen in liquid N_2_ and kept at −80°C prior to RNA extraction. We used the Qiagen RNeasy Bacteria Mini Kit for isolation of RNA samples according to manufacturer's instructions.

To extract bacterial RNA from infected plants we used a protocol for extracting RNA from *P.syringae* recovered from infected leaves as described by Yu *et al*. [55]. RNA quantification was performed with a UV-VIS UNICAM Model Heλiosβ spectrophotometer. Reverse transcription was carried out from 1 µg of total RNA for gene expression analysis, by using OMNISCRIPT (Qiagen) and random hexamer primers (Promega).

### Expression analysis by qRT-PCR

Specific primers for real-time PCR were designated by using Primer3 program according to the sequence of the corresponding gene. Primers used for the assay are described in Table 1. 4 µl of diluted cDNA were used per 25 µl qPCR Quantitative real-time PCR was performed with SybrGreen Premix ExTaq (Takara) in a Cepheid Smart Cycler system Relative levels of the monitored genes were normalized with *recA* and used as an internal reference.

**Table 1:** Primers used for Real-Time PCR.

Gene	Funtion	Primer
*cfa1*	CFA synthesis	F 5′-AAAACCATCGTCGACATTCTG-3′
		R 5′-GTTGGCGTTGAGGTCGATA-3′
*cmaB*	CMA synthesis	F 5′-AATTCGACACCCGACAAGAC-3′
		R 5′-ACTAGGGGCTTCAGGTCCAT-3′
*cfl*	CMA and CFA union	F 5′-ACAGCTGAAGCAGCACTTGA-3′
		R 5′-CGAGGATCTCTCGGTAGTCG-3′
*hrpL*	Transcription factor σ^L^	F 5′-TCTCCAGTGCGTGTTTCTTG-3′
		R 5′-AGCTTTCCTGATACGGCTGA-3′
*hrpA*	*hrp* pilus	F 5′-CCTCCAAACTCACCAACCTT-3′
		R 5′-CGGACTCTTTACTGGCCTTG-3′
*avrPtoB*	AvrPtoB type III effector	F 5′-ACCCTATCGCGTCACAATTC-3′
		R 5′-CATGAACGCCAGGTCCTTAT-3′
*psyI*	AHL synthase	F 5′-GGCTTGAATGGAATGTTCGT-3′
		R 5′-CAGGTGTTGATCAGCCGTAA-3′
*psrA*	Sigma regulator of *psyI*	F 5′-AGACGCATAACCTGCTCGAT-3′
		R 5′-ACATGACGCTGGTCAATGAA-3′
*cma X*	Magnesium transporter	F 5′-AGCGGGACATTTATGGTCAG-3
		R 5′-CTGTTGTTCAGCTCGTTCCA-3
*recA*	Internal reference	F 5′-CGGCAAGGGTATCTACCTCA-3′
		R 5′-CTTTGCAGATTTCCGGGTTA-3′

### Statistical analysis

Statistical analysis was carried out using a one-way analysis of variance in the Statgraphics-plus software of Windows V.5 (Statistical Graphics Corp., Rockville, MD, USA). The means were expressed with standard errors and compared using a Fisher's least-significant difference test at the 95% confidence interval. All the experiments were repeated at least three times.

## Results

### 1. Analysis of bacterial behaviour *in vitro*

#### 1.1 Antibacterial activity of Hx

**Figure 1:**
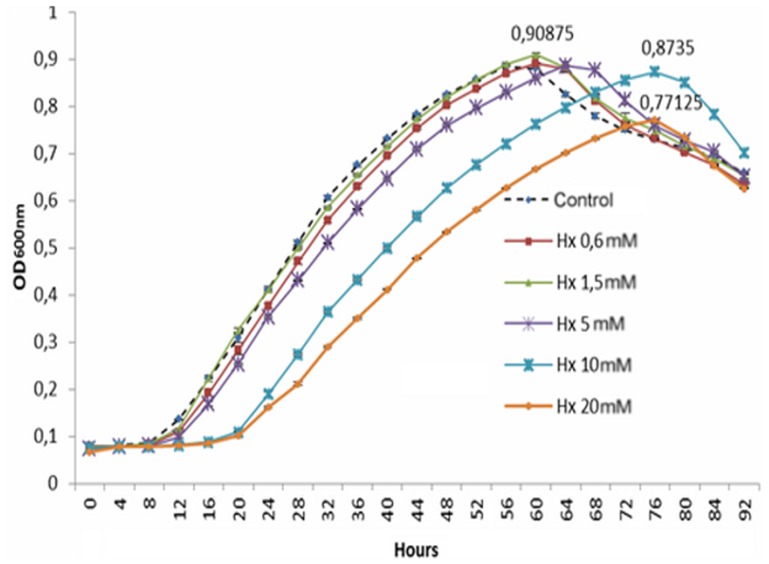
Influence of different hexanoic acid (Hx) concentrations on the growth rate of *Pst* DC3000. The results are presented as growth curves (in hours) of *Pst* DC3000 during its culture in the presence of 0.6, 1.5, 5, 10, and 20 mM Hx. The optical density at 600 nm was measured at 10-min intervals. The results represent the means with standard deviation.

The growth of *Pst* DC3000 in the absence or presence of different concentrations of hexanoic acid (Hx) (0.6, 1.5, 5, 10, and 20 mM) was tested in LB medium. Higher concentrations were not used because damage to the tomato plants in our experimental assays was observed at concentrations higher than 20 mM. The bacterial growth curves were generated using a BioScreen system for 96 h (Fig. 1), and the results show that Hx at concentrations of 10 and 20 mM promoted slow bacterial growth and that the lag phase was markedly longer in these samples compared with those exposed to lower concentrations (20 and 8 h, respectively). Moreover, Hx at a concentration of 20 mM produced an inhibition of 15% in bacterial growth. The addition of Hx at concentrations of 0.6, 1.5, and 5 mM did not reduce the growth of the target compared with the control.

We can conclude that Hx at a concentration of 10 and 20 mM could have a temporary bacteriostatic effect against *Pst* DC3000 because at these concentrations the lag phase lasts longer. However a bactericidal effect cannot be ruled out, at these concentrations.

#### 1.2 Analysis of Hx bactericidal effect

**Figure 2:**
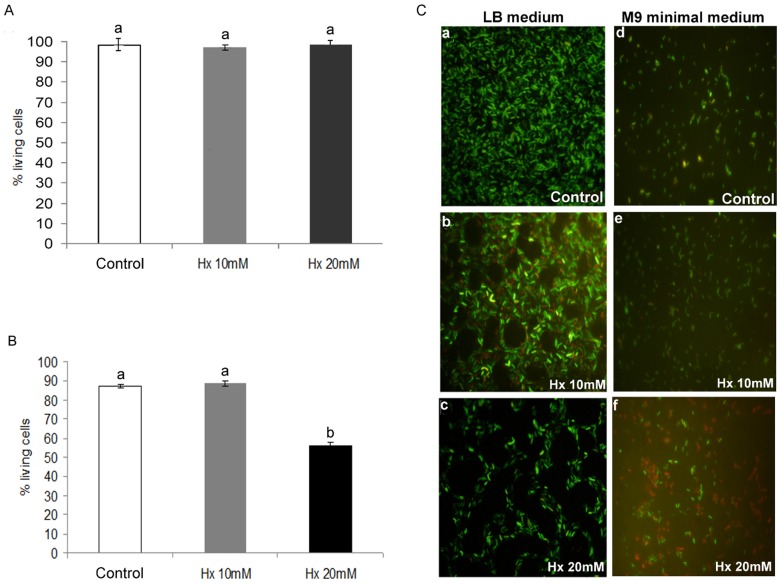
Bactericidal effect of Hx treatment at concentrations of 10 and 20 mM on *Pst* DC3000. Long-term effect of Hx in LB medium (A). Short-term effect of Hx in minimal medium (B). The charts illustrate the percentage of live cells, which was determined using a Live/Dead Bacterial Viability Kit. The images show the viable (green) and dead (red) cells and illustrated that aggregates are formed after exposure to Hx concentrations of 10 mM and 20 mM only in the long-term experiments (C.2, C.3).

The bactericidal effect of Hx was investigated by labelling the bacteria with the Live/Dead Cell Viability Assay kit, a two-colour fluorescence assay based on membrane integrity. The bactericidal effect was assessed using bacteria incubated for 96 h in LB medium with an Hx concentration of 10 and 20 mM (based on the results described above) and compared to that obtained with the untreated control. A very low number of dead bacteria (red labelled) were observed both in the untreated control and in the groups with 10 and 20 mM of Hx, and no significant differences were observed between the groups (Fig. 2A). Moreover, the non-treated controls had higher concentrations of living bacteria (Figs. 2Ca, 2Cb, and 2Cc). The presence of dead bacteria in the untreated controls appeared to be due to the experimental conditions used for growth, i.e., nutrient availability was a limiting factor due to the higher concentration that reached the control bacteria. Thus, Hx at concentrations of 10 and 20 mM caused no statistically significant changes in bacterial viability over a long period of time. This result may be because the bacteria enter the viable but non-culturable stage (VBNC) (Fig. 2Cc). In addition, at these concentrations, we microscopically observed the formation of multicellular aggregates that were not observed in the controls and the other treatment groups (Fig. 2Ca), and these aggregates were better defined in the group treated with 20 mM Hx (Fig. 2C.c).

Taking into consideration these results, the next question we addressed was whether the direct effect of Hx at 10 and 20 mM on the bacteria is a short-term bactericidal effect, which implies a reduction in the initial bacterial population followed by its recovery over the long term. To test this, the bactericidal effect was tested in bacteria that were incubated for 16 h in M9 minimal medium with Hx at concentrations of 10 and 20 mM. As shown in Figure 2B, a low number of dead bacteria were observed both in the untreated control bacteria and in the bacteria incubated with Hx at 10 mM. However, the bacteria incubated with Hx at 20 mM presented a clear short-term bactericidal effect of Hx because the number of nonviable cells represents 44% of the total number of cells (Figs. 2Cd, 2Ce, and 2Cf).

Taking together all of these results, we can conclude that the Hx effect on the target bacteria is dependent on the concentration: Hx at 10 mM has a temporary bacteriostatic effect, whereas Hx at 20 mM has a short-term bactericidal effect on half of the cells, which may explain the partial recovery of population growth over the long term.

#### 1.3 Hx affects bacterial gene expression *in vitro*

**Figure 3:**
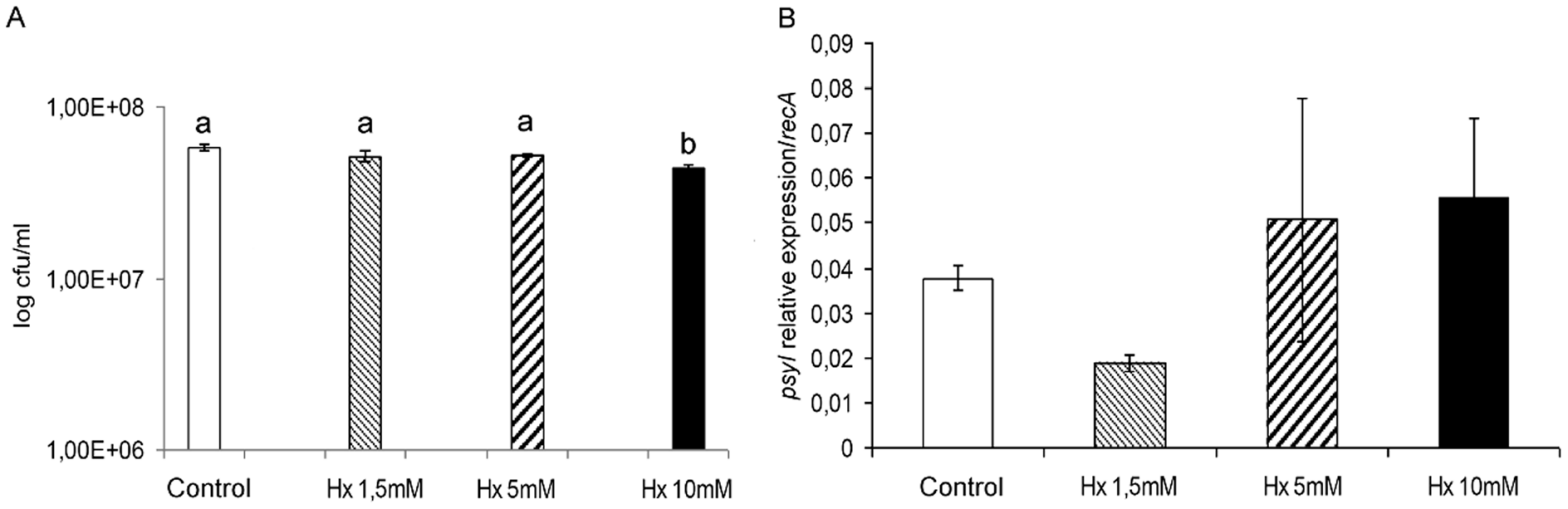
Effect of Hx on bacterial growth and QS-related genes. The bacteria were grown in LB medium and LB plus Hx at 1.5, 5, and 10 mM for 18 h and then plated on KB agar plates to count the colony forming units (A). The transcript levels of *psyI* were determined by qRT-PCR. The relative expression levels of *psyI* were normalized to those of *recA* (B). The results represent the means and standard deviations from three different experiments.

We also performed *in vitro* assays to determine the Hx effect on bacterial gene expression. To achieve this goal, we selected genes related to the pathogenesis and survival of *Pst* DC3000, such as quorum sensing establishment-related genes (*psyI*), coronatine synthesis-related genes (*cfa1*, *cmaB* and *cfl*), genes selected as representative of the type III secretion system and the type III secretion system-associated pilus, respectively (*hrpL* and *hrpA*), and genes responsible for effector synthesis (*avrPtoB*). Hx was added at different concentrations (1.5, 5, and 10 mM) to the medium, and the bacteria were cultivated in the Hx-supplemented medium for 18 h at 28°C. After 18 h of incubation, we observed a significantly lower bacterial concentration in the group treated with 10 mM Hx (Fig. 3A).

The monitoring of the expression of *psyI* revealed very low levels in both the controls and the bacteria incubated with different concentrations of Hx. The expression of this gene was slightly higher in the bacteria incubated with Hx at 5 and 10 mM. However, at 1.5 mM Hx, the expression of this gene was lower (Fig. 3B).

**Figure 4:**
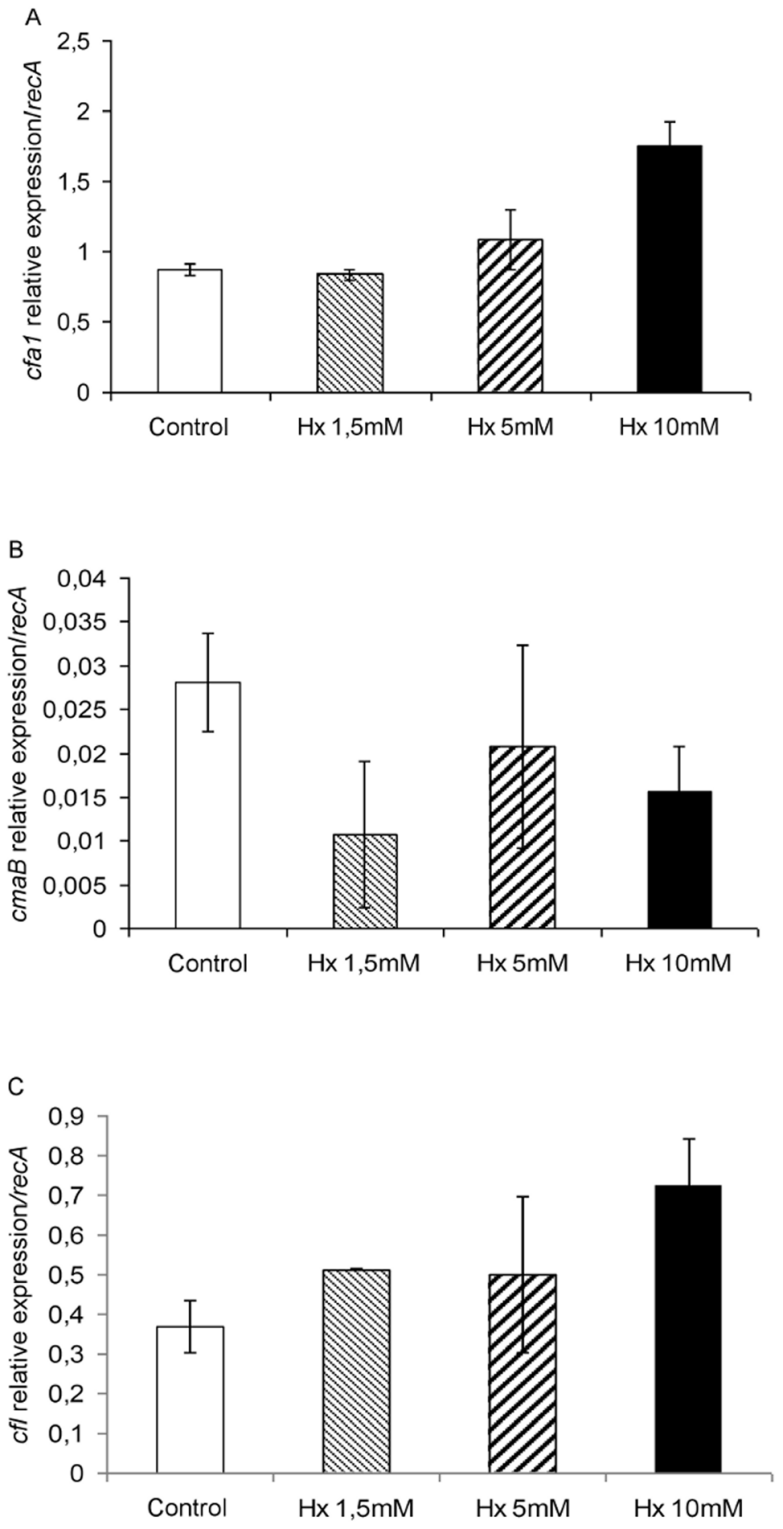
Relative expression of COR synthesis genes (*cfa1* (A), *cmaB* (B), and *cfl* (C)) during bacterial growth in the presence of different Hx concentrations. Template cDNAs were generated from the total RNA extracted from bacteria grown as described in Figure 3. The *recA* gene was used as an endogenous reference gene. The results represent the means and standard deviation from three different experiments.

The transcript levels of *cfa1* and *cfl* (Figs. 4A and 4C) were induced in bacteria grown with Hx at a concentration of 10 mM compared with those grown without Hx or with 1.5 and 5 mM Hx. However, the *cmaB* (Fig. 4B) expression level was very low, and practically equal amounts of its transcripts were detected in all of the samples or slightly lower amounts were detected in the presence of Hx.

**Figure 5:**
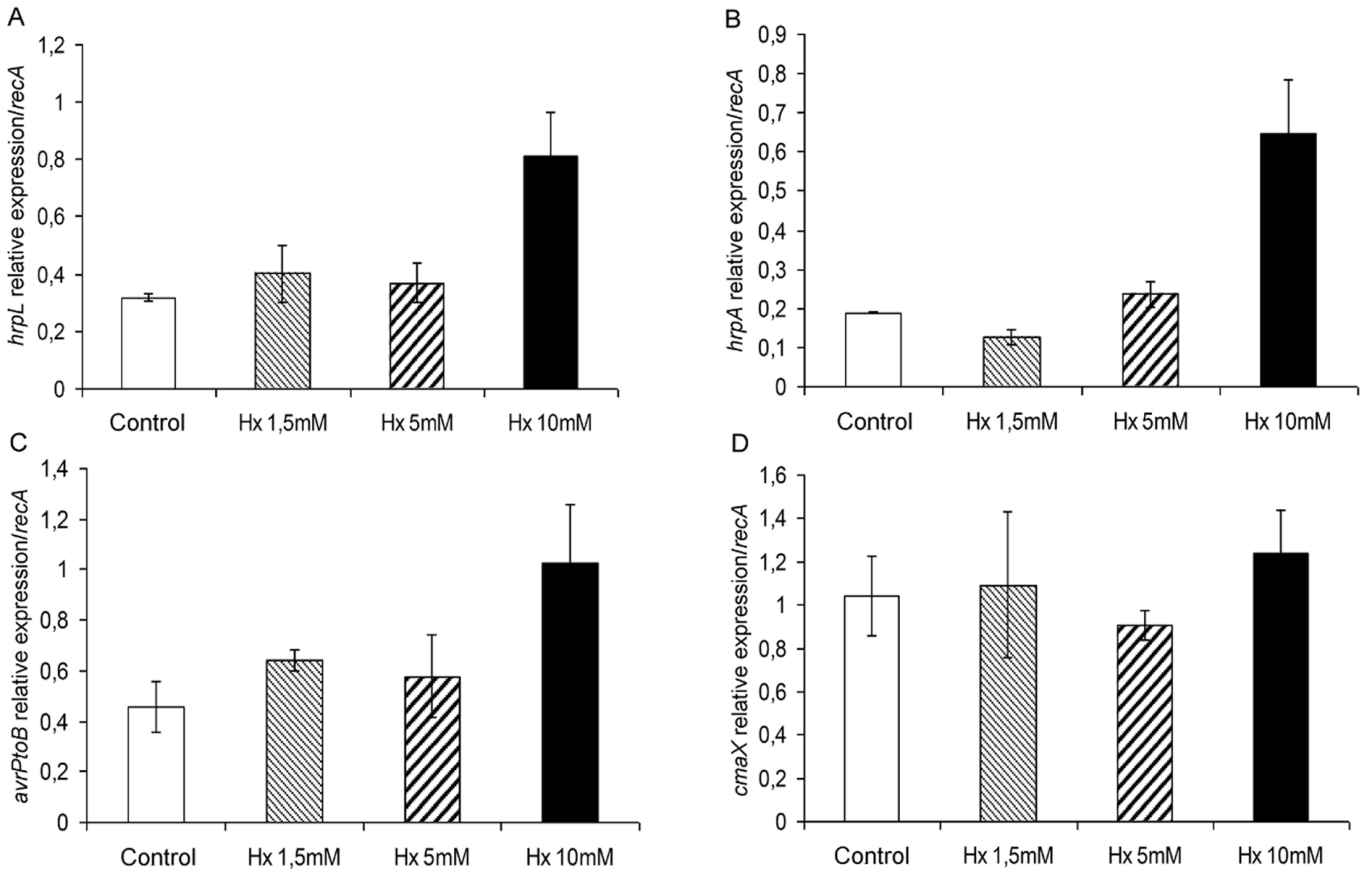
Expression of type III secretion system- and other virulence factor-related genes in response to different hexanoic acid concentrations. The transcript levels of *hrpL* (A), *hrpA* (B), *avrPtoB* (C), and *cmaX* (D) were examined as described in Figure 3, and the *recA* gene was used as an internal reference. The results represent the means and standard deviation from three different experiments.

Similar results to those obtained for *cfa1* and *cfl* were observed in the analysis of *hrpL, hrpA*, and *avrPtoB* (Figs. 5A, 5B, and 5C). In contrast, the analysis of the expression of *cmaX*, a CorA-like Mg^2+^ transporter, revealed similar levels of expression regardless of the concentration of Hx (Fig. 5D).

Taken together, these results demonstrate that the expression of the pathogenesis-related genes *cfl, cfa1, hrpL, hrpA,* and *avrPtoB* is enhanced with exposure to Hx at a concentration of 10 mM, which also affected bacterial growth. In contrast, the *cmaB* and *cmaX* expression levels were essentially equal regardless of the Hx concentration. These results indicate the involvement of the pathogenesis system in bacterial survival under abiotic stress conditions.

To confirm this hypothesis, the behaviour of mutants defective in these genes (*ΔhrpL*, *ΔhrpA*, *Δcma-cfa*, and *Δpto* (*ΔAvrPto-ΔAvrPtoB*)) was analysed under the same conditions to test the antimicrobial activity of Hx. Each mutant showed a similar level of survival to *Pst* DC3000, regardless of the Hx concentration (data not shown).

### 2. Analysis of bacterial behaviour *in planta*

**Figure 6:**
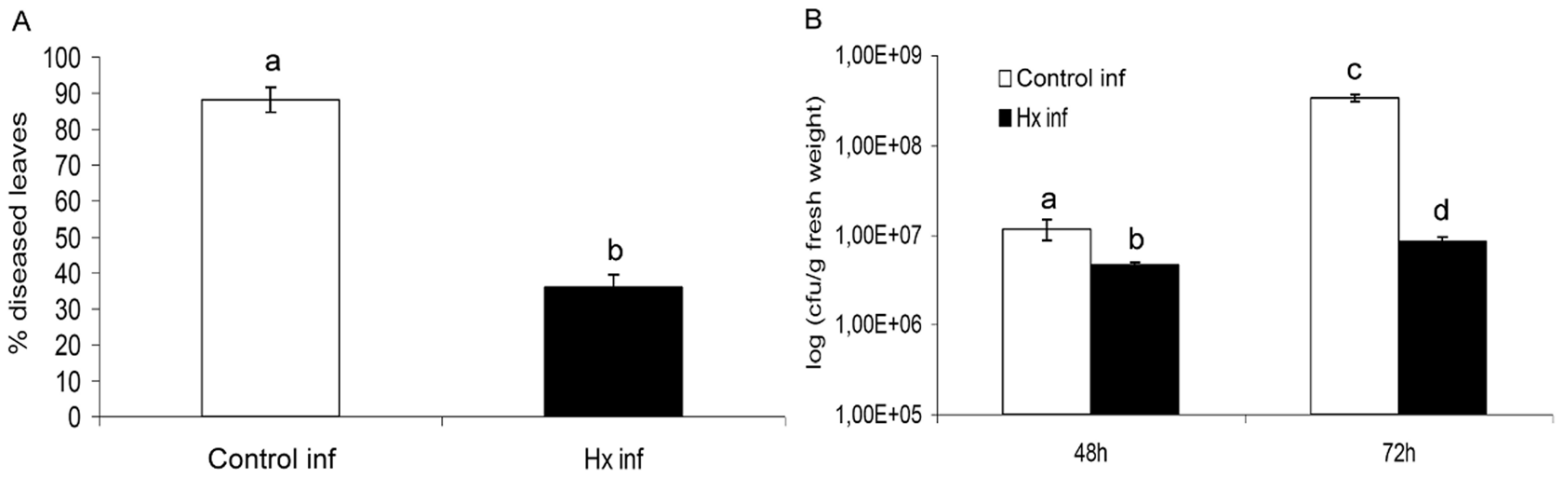
Hexanoic acid-induced resistance against *Pst* DC3000. Four-week-old tomato plants were treated with Hx through soil drench and dip inoculated with *Pst* DC3000. Seventy-two hours after inoculation, the disease rating was scored by measuring the percentage of infected leaves in relation to the total number of analysed leaves (A) and by recounting the bacterial populations through plating in agar-King's B medium (B). The data show the average values ± standard error (*n* = 20). The different letters represent statistically significant differences (*P*<0.05; least-significant difference test).

Assays were conducted under the conditions described by Scalschi *et al.* [4]. The efficacy of Hx treatment was confirmed at both 48 hpi and 72 hpi by the reduction in disease symptoms and the reduction in the number of colony-forming units observed in the treated plants compared with the untreated plants, and major differences were observed at 72 hpi (Figs. 6A and 6B). Furthermore, we examined the expression of the genes analysed above after the infection of tomato plants with *Pst* DC3000 to determine whether their expression was altered by the treatment.

#### 2.1 QS is affected by Hx treatment

The gene expression was evaluated 2, 4, 10, 18, 48, and 72 h after infection. At the early stages, due to the low bacterial concentration, the obtained values exhibited a broad range, and we thus selected the 48- and 72-h time points for further analyses. At these time points, the greatest changes in the defence system of the treated plants were induced against *Pst* DC3000 [4].

**Figure 7:**
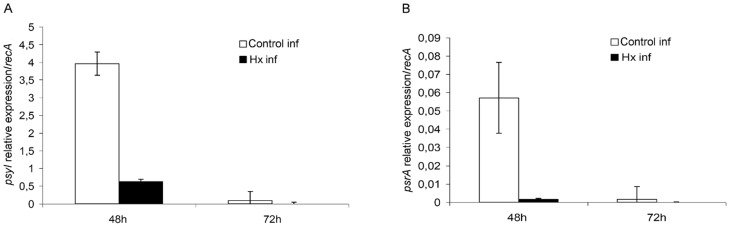
Effect of hexanoic acid treatment on quorum sensing establishment. The relative expression of *psyI* (A) and *psrA* (B) was determined by qRT-PCR during the infection of treated and untreated tomato plants with *Pst* DC3000. The relative expression levels of *psyI* and *psrA* were normalized to those of *recA*. The results represent the means and standard deviation from three different experiments.

The epiphytic phase of *Pst* DC3000 is considered one of the first steps in the infection process [30], [31]. Thus, the transcriptional activity of the *psyI* and *psrA* genes was monitored during the infection of treated and untreated tomato plants. As illustrated in Figure 7A, the expression of *psyI* was significantly lower in the bacteria from the treated plants at 48 hpi. Although the *psyI* expression level was decreased in bacteria from both untreated and treated plants at 72 hpi, the difference between these bacteria was maintained.

In contrast, the monitoring of the relative expression of *psr*A revealed very low levels of expression at both 48 and 72 hpi. However, its expression was higher in the bacteria from untreated plants at 48 hpi (Fig. 7B).

#### 2.2 Expression of COR synthesis genes is reduced by Hx treatment

**Figure 8:**
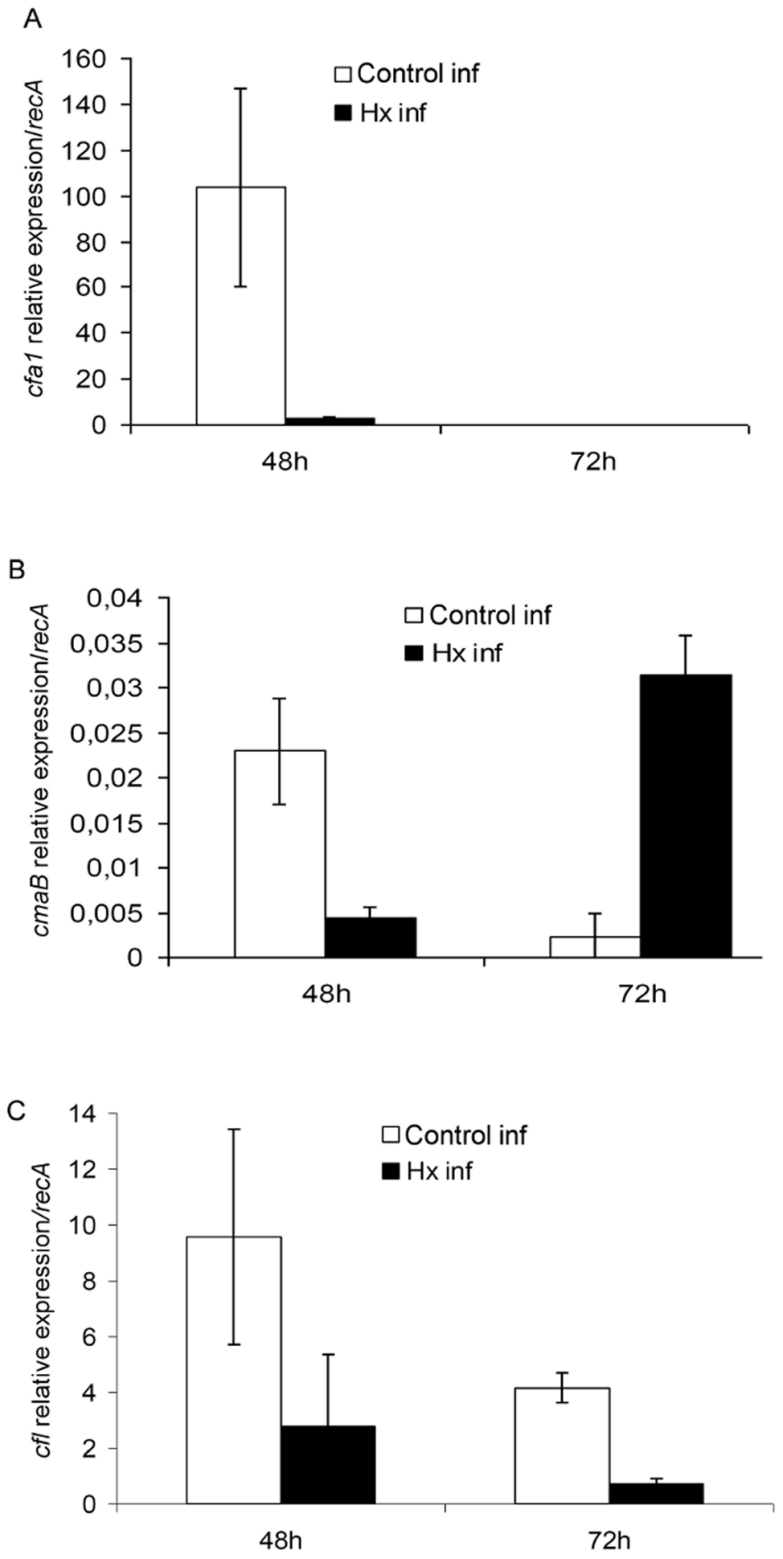
Hx acid treatment reduces expression of COR synthesis genes. The relative expression level of *cfa1* (A), *cmaB* (B) and *cfl* (C) were measured at 48 and 72 h postinoculation, using qRT-PCR with specific primers. The gene expression was normalized with *recA* that was used as endogenous references. The results represent the means of three different experiments with standard deviation.

To investigate the effect of Hx treatment on *Pst* DC3000, the expression levels of the coronatine synthesis genes *cfa1*, *cmaB*, and *cfl* were measured in bacteria extracted from both treated and untreated tomato plants and were compared with those obtained in culture media (Fig. 8). As expected, the transcription levels of *cfa1* and *cfl* were increased at 48 hpi and were higher in the bacteria from untreated plants compared with those from the treated plants. Although the expression of *cfl* was significantly decreased in the bacteria from the untreated plants at 72 hpi, its transcription levels exceeded those obtained in the bacteria from the treated plants. In contrast, *cfa1* expression was hardly detected at this time point (Figs. 8A and 8C). A similar increase was observed with *cmaB* at 48 hpi, but elevated levels in its expression were recorded in the bacteria from the treated plants at 72 hpi (Fig. 8B).

#### 2.3 Type III secretion system is affected by Hx treatment

**Figure 9:**
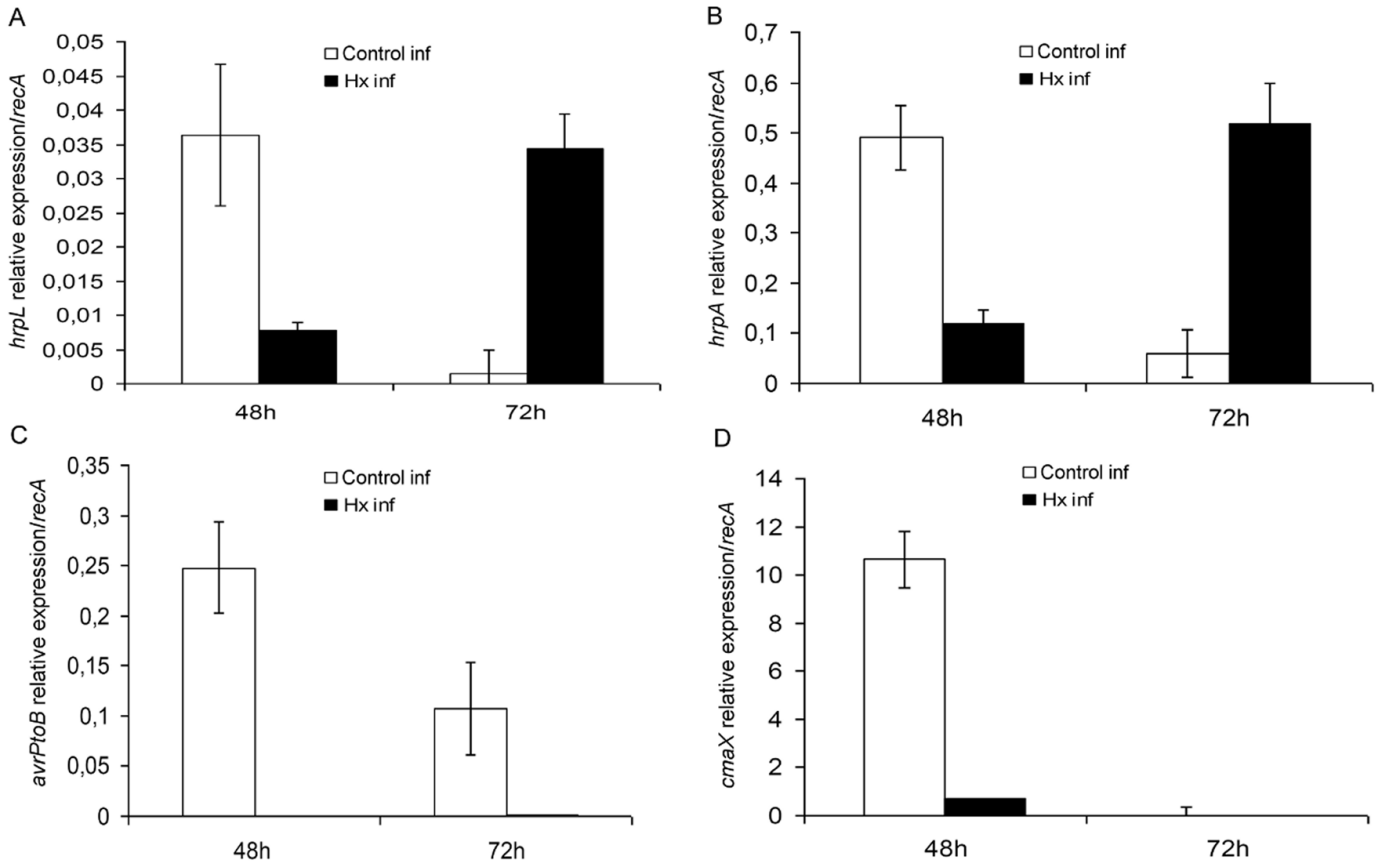
Effect of Hx acid treatment on Type III secretion system and other virulence factors. The transcript levels of type III secretion system associated genes (*hrpL* (A), *hrpA* (B), *avrPtoB* (C)) and CorA-like Mg^2+^ transporter (*cmaX* (D)) was determined at various time points after infection of tomato plants with *P.syringae* DC3000.The *recA* gene was used as endogenous references. The results represent the means of three different experiments with standard deviation.

We assayed the expression of the *hrpL* and *hrpA* genes, which were selected as representative of the type III secretion system and the type III secretion system-associated pilus, respectively, and the expression of the *avrPtoB* gene, which is responsible for effector synthesis (Fig. 9).

To our surprise, we did not observe a strong induction of *hrpL* upon infection; on the contrary, we found that *hrpL* was induced to higher levels *in vitro* than *in planta*. Although Hx treatment inhibits the expression of this gene at 48 h, its expression was enhanced in the treated plants compared to the untreated ones at 72 hpi (Fig. 9A).

In contrast, the expression of *hrpA*, the major component of the type III secretion system-associated pilus, was stronger in the infected plants than *in vitro*, with the exception of the bacteria incubated with Hx at 10 mM, which exhibited similar expression levels to those observed in the infected plants. *In planta*, this gene has a similar behaviour to that observed with *hrpL* (Fig. 9B).

Furthermore, the expression of the gene involved in the synthesis of the specific effector AvrPtoB was monitored (Fig. 9C). At both 48 and 72 hpi, substantial increases in the *avrPtoB* transcription levels were recorded in the untreated plants compared with the treated plants.

#### 2.4 Other effects of Hx acid treatment

The relative expression levels of the genes related with the CorA-like Mg^2+^ transporter, namely *cmaX* and *corA*, were measured in bacteria recovered from treated and untreated plants (Fig. 9D). At 48 hpi, the transcription level of *cmaX* was decreased by more than 10-fold in the bacteria recovered from treated plants.

#### 2.5 Is *Pseudomonas* able to overcome Hx-IR over time?

The findings that the bacterial populations in treated plants increase even though fewer symptoms are observed and that some of the pathogenesis-related genes are expressed at 72 h led us to ask whether the bacteria can overcome the induced defence mechanisms in plants. To study the long-term bacterial behaviour, similar experiments were conducted over a 30-day period.

**Figure 10:**
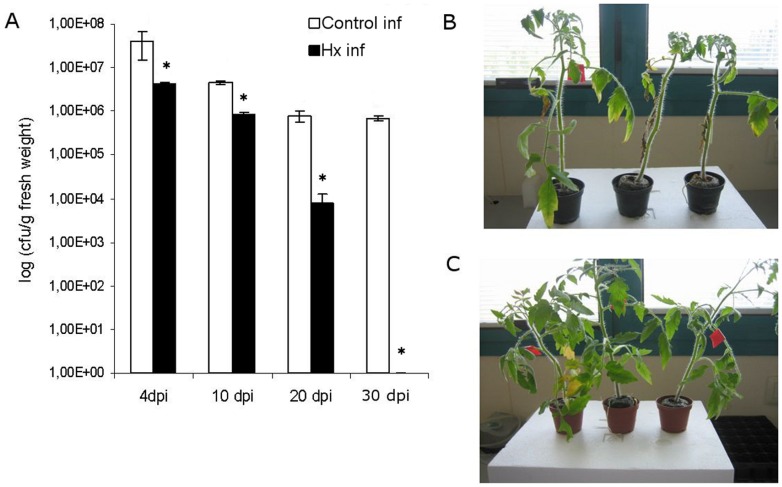
Hx treatment has a long lasting effect. Plants were treated with 0.6 mM Hx and dip inoculated. The bacterial growth was evaluated at 4, 10, 20 and 30 days after inoculation with *P.syringae* DC3000 (10^5^ cfu/ml) (A). The pictures of representative untreated (B) and Hx treated (C) plants were taken 20 days after inoculation.

After 20 days, an increase in disease severity followed by abscission of the infected leaves was observed in the untreated plants. The treated plants presented a better physical condition than the untreated ones, as determined by the lack of extension of infection to the younger uninoculated leaves and the absence of leaf abscission (Figs. 10B and 10C).

The bacterial growth was higher in the untreated plants than in the treated ones. Although the bacterial populations were increased in both the treated and non-treated plants after four days, at longer time points, the number of pathogens was markedly decreased in the treated plants. In fact, in most of the samples, the bacterial population was under the limit of detection (Fig. 10A).

## Discussion

In previous works, we demonstrated that Hx presents antifungal activity against the phytopathogen *B. cinerea in vitro* at nonphytotoxic concentrations [1]. Similar studies were performed by other researchers who evaluated the *in vitro* effect of several inducers on the fungus *Mycosphaerella fijiensis* [32], but little is known about the effect of resistance inducers on bacteria. Previously, we demonstrated the efficacy of Hx as a plant defence inducer against *Pst* DC3000, and we studied the molecular and physiological mechanisms behind Hx-IR [4]. To elucidate the details of the mechanism of action of Hx, the present study was focused on bacterial response both to the direct effect of Hx, which was applied *in vitro* to the culture medium, as to the changes observed in the plant defence mechanisms in response to Hx, which was applied as an inducer agent.

The main findings of the *in vitro* experiments were the following: Hx at 10 mM has a bacteriostatic but not bactericidal effect against *Pst* DC3000, whereas 20 mM Hx exerts an initial and partial bactericidal effect, which may explain the lower final concentrations of bacteria observed in the long-term experiments.

Furthermore, the formation of bacterial aggregates at these concentrations may be correlated to the adaptation of the bacteria to the stress conditions generated by the action of Hx at high concentrations, may explain the slow growth or the entry into the VBNC stage of the cells, and may be considered a mechanism through which the bacteria protect themselves under stress conditions, as reported for *Erwinia amylovora* [33] and *Ralstonia solanacearum* [34]. The bacterial counts obtained using the Live/Dead Kit are directly related to the real state of the bacteria and not only to their ability to form colonies in solid medium.

These results are further supported by the gene expression analysis of virulence- and quorum sensing-related genes, which revealed that there was no effect of Hx at concentrations lower than 10 mM. The COR synthesis genes, with the exception of *cmaB*, were relatively over expressed when Hx was added at a concentration of 10 mM to the medium. Another aspect that must be taken into consideration is that the analysis of these genes was performed in the exponential growth phase. The higher expression of *cfa1* compared to that of *cmaB* is in agreement with the results of other researchers, who observed that CFA is released with coronatine in the culture medium [35]. Moreover, previous studies performed by other researchers revealed that COR production is regulated by temperature and that the expression of *cma* genes increases during the exponential growth phase while their expression is temperature independent during the stationary phase [36]. The same researchers also reported that the activity of *cma* expression is dependent on the genetic background of the host strain.

In contrast, direct contact with the host did not appear to be required for *hrpA*, *hrpL*, and *avrPtoB* expression because their level of expression in the *in vitro* controls was similar or even exceeded those observed *in planta*. These results are supported by the findings reported by Haapalainen *et al*. [37]. Moreover, the increase in *hrpA*, *hrpL*, and *avrPtoB* expression due to exposure to Hx at 10 mM may be attributed to the abiotic stress caused by higher concentrations of Hx and indicates an important role for T3SS in bacterial adaptation and survival. Rahme *et al*. [38] showed that the *hrp* gene expression in *P. syringae* pv. phaseolicola is under the control of multiple physiological and environmental factors, specifically pH, osmotic strength, and catabolite repression. According to these authors, the optimal expression of all *hrp* genes *in vitro* is obtained under stress conditions that stimulate the environment of the leaf apoplast with respect to a low osmolyte content and pH 5.5. Similar results were obtained by van Dijk *et al*. [39], who demonstrated that T3SS is environmentally regulated at both the transcriptional and post-transcriptional levels and that environmental conditions, such as temperature, and pH play an important role in both gene expression and protein secretion. In contrast, the increase in T3SS-associated gene expression may be related to the growth pattern. Taking into consideration that at the lag phase lasts longer at this Hx concentration, the expression of these genes at 18 h may be required for the bacteria to pass from the lag phase to the exponential phase. Recently, Lee *et al*. [40] revealed the importance of the T3SS and specific effectors for the development of aggregates on the leaf surface. Thus, the increase in the transcription levels of genes that encode the structural and regulatory proteins associated with T3SS may be correlated with the aggregate formation observed after exposure to 10 mM Hx. Additionally, the elevation of the relative expression of *psyI* at this concentration supports our results because elevated levels of the quorum-sensing signal molecule AHL result in the expression of virulence factors that may be necessary for bacterial adaptation and survival under stress conditions.

To investigate the involvement of these factors in bacterial adaptation to stress conditions and survival, we used different mutants with impairments in the analysed genes. Although the mutants did not show reduced survival, the activation of these genes may contribute but not be essential for survival, and their involvement in bacterial adaptation cannot be ruled out. Because *P. syringae* has numerous other effectors, one or more of these may fulfil this role.

Regarding the changes that *Pst* DC3000 undergoes *in planta*, the study of gene expression patterns related to virulence or quorum-sensing establishmen *in planta* may help illuminate how the bacteria can survive and overcome plant defences and, in our case, how the bacteria responds to the induced plant defences since previous works described the main signalling pathways that are involved in basal and induced resistance to this pathogen. The study of hormonal patterns and the expression of marker genes revealed that the activation of these pathways occurs starting 48 hpi [4], which is correlated with transcriptional changes in bacterial genes and disease onset.

The dip-inoculation method used in our studies forces the bacteria to enter plants through natural openings present on the plant surface to establish infection sites within the apoplast. The fact that the treatment lost its effectiveness when the plants were infected by vacuum infiltration [4] indicates the possibility that the treatment may act in the early stages of bacterial infection, such as entry into host tissue and bacterial colonization of the intercellular spaces between plant cells.

The results obtained from our study of the factors involved in the early stages of infection, such as *psyI* and *psrA*, point to the fact that QS is reached earlier in the untreated plants compared with the treated ones, thereby delaying its establishment. It is known that QS in bacteria regulates the gene expression of virulence factors. Thus, this delay can be added to an activation of plant defences that may prevent or at least mitigate pathogen attack. Moreover, Chatterjee *et al*. [41] demonstrated that *psrA* has a positive effect on disease because the mutation of this gene affects the time of the onset of symptoms and their severity. These phenotypes correlate with the observed reduced expression of *hrpL*, which encodes an alternate sigma factor. Thus, in addition to delaying the establishment of QS, Hx treatment may diminish *Pst* DC3000 virulence by reducing the *PsrA* expression levels.

In previous works, we found that Hx treatment affects the action of COR by preventing stomatal reopening after bacterial infection [4]. One of the purposes of this study was to establish whether the changes in the infected plants due to Hx treatment could produce an alteration in the expression of genes involved in the synthesis of this molecule. Interestingly, we observed that Hx treatment reduces the expression of the COR synthesis genes *cfa1*, *cmaB*, and *cfl* at 48 hpi. This finding has an important implication in the enhanced resistance of treated plants, which presented markedly reduced disease severity compared with the untreated plants, because COR has been shown to suppress plant defence responses [23]. In contrast, at 72 hpi, elevated *cmaB* expression levels were recorded in the treated plants. Surprisingly, we also observed an increment in *hrpL* expression at this time point. According to Sreedharan *et al*. [42], HrpL modulates *cfa1* and *cmaB* expression. It is not clear why this increase occurs only in the case of *cmaB*, although it is possible that the bacteria have an excess of CFA due to the higher expression levels of *cfa1* at 48 hpi, which could result in the synthesis of COR. This would therefore involve a delay in symptom development, which was not observed in the long-term experiments. Thus, this discrepancy in the synthesis of the two components of COR in treated plants leads to a delay of disease onset and may influence the survival of the bacteria. On the other hand, the transcription of these genes in infected plants was extensively induced compared with that recorded in medium, which suggests that host signals are involved in their full activation [43].

Another factor that is important in the pathogenicity of *P. syringae* is the type III secretion system, which is encoded by the *hrp/hrc* regulon. In general, *hrp* genes are induced in plants and minimal nutrient media but repressed in complex media. Their expression is influenced by carbon and nitrogen sources, pH, osmolarity, temperature, and plant signal molecules [44]. In the bacteria from treated plants, a delay in *hrpL* and *hrpA* expression was observed. Although the expression of *hrpL* was 10-fold higher *in vitro* than *in planta*, other researchers have clearly shown that T3SS requires HrpL for its function [45]; however, the low expression levels observed *in planta* may be an indication of the existence of additional levels of regulation that have not been previously described. These results may also indicate that HrpL does not accumulate to high intracellular levels, suggesting that some type of feedback regulation could take place between the secretion and production of HrpL, perhaps through the degradation of intracellular HrpL [37].

In *Pst* DC3000, the T3SS is induced when the bacteria enter the leaf mesophyll through stomata. This bacterium can control the volume of type-three protein secretion by the expression level of HrpA pilin and the number of pili formed on the cell surface. The amount of HrpA protein needed per individual T3SS machinery depends on the distance between the bacterium and the plant cell [46]. It is possible that Hx treatment delays the contact of the bacterium with the plant cells and that this delay is responsible for the elevated levels of *hrpA* transcripts observed later in the treated plants.

Given that the current view of molecular plant-pathogen interactions is that pathogens secrete effectors into the apoplast and/or cytoplasm of host plants to suppress host defences, facilitate colonization, and trigger symptom production [47], [48], several effectors have been studied. AvrPtoB has been found to act inside plant cells to suppress programmed cell death associated with plant immunity and increases pathogen growth, dissemination, and disease formation [19], [49]. Once infection is established, this effector induces host programmed cell death to gain access to nutrients or aid pathogen dissemination. The fact that Hx treatment diminishes *avrPtoB* expression *in planta* is supported by the results reported by Abramovitch and Martin [49] because the symptoms are greatly reduced, even though the size of the bacterial population is increased to a lesser extent compared with untreated plants.

The virulence of pathogenic bacteria is also affected by divalent cations, of which Mg^2+^ plays a central role. CorA or CmaX is the major Mg^2+^ transporter. The importance of CorA or CmaX in virulence was previously reported [26], [50]. We observed that Hx treatment clearly reduces *cmaX* expression, and this decrease may affect the production of several plant cell wall-degrading exoenzymes, such as pectate lyase, polygalacturonase, cellulose, and protease, which are responsible for the maceration of plant tissue. This reduction could also affect bacterial survival and multiplication *in planta*. Our data are supported by the results obtained by Kersey *et al*. [51] *in planta*. These researchers noted a significant reduction in the cfu of the CorA^−^ mutant recovered from infected tissue compared with the CorA^+^ parent.

Because several genes were found to be expressed in the bacteria from the treated plants at 72 hpi, we then investigated whether the bacterial population could increase to a point where the bacteria might overcome the plant defence responses. The results obtained in the long-term assays discard this hypothesis and point to the induction of defence as a control strategy that reduces the pathogenicity by limiting the rate of pathogen multiplication [52] and by changing the expression pattern of virulence-related genes.

The conclusions we can draw for the effects of Hx on bacteria are that Hx has a direct *in vitro* effect on bacteria at high concentrations and that its effect on treated plants demonstrates that it acts as an inducer of resistance. In the latter case, the Hx effect is not conditioned by its presence in the plant, as demonstrated by our previous finding that Hx does not accumulate in treated plants because a chromatographic analysis revealed no detectable trace of either Hx or 13C-Hx above the detection limits (10 µg L^−1^) [6]. Thus, one of the most interesting outcomes of this study is that the two effects are opposite: the direct effect of Hx consists of the activation of the bacterial genes involved in virulence, and its effect as an inducer of resistance is based on the inactivation of these genes, a delay in their expression, or a discrepancy in their activation that leads to the development of less symptoms and the decreased ability of the bacteria to grow and may even compromise its long-term survival. It is interesting to note that the transcriptional changes in the bacterial genes in the treated plants occur at the same time point (48 hpi) as the changes in the plants. Additionally, the differences in the bacterial population size are more pronounced between the treated and non-treated plants at 72 h. Because most of the bacterial genes studied are repressed at 48 h, this repression apparently prevents bacterial growth. This finding suggests that the pathogenesis and survival of the bacteria is affected by plant response when Hx is applied as a resistance inducer and that Hx affects both aspects of the molecular “arms race” (i.e., the plant immune system and pathogen virulence effectors) [53] to benefit the plant immune system.

## Supporting Information

File S1 **File S1 includes the following: Data S1**. Table presenting data underlying the results described in figure 1. **Data S2.** Table presenting data underlying the results described in figure 2. **Data S3.** Table presenting data underlying the results described in figure 3. **Data S4.** Table presenting data underlying the results described in figure 4. **Data S5.** Table presenting data underlying the results described in figure 5. **Data S6.** Table presenting data underlying the results described in figure 6. **Data S7.** Table presenting data underlying the results described in figure 7. **Data S8.** Table presenting data underlying the results described in figure 8. **Data S9.** Table presenting data underlying the results described in figure 9. **Data S10.** Table presenting data underlying the results described in figure 10.
(DOC)
