# Supplementary material for: Resistance Inducers Modulate Pseudomonas syringae pv. Tomato Strain DC3000 Response in Tomato Plants
Source: PLoS One. 2014 Sep 22;9(9):e106429. doi: 10.1371/journal.pone.0106429 (PMC4171367; doi:10.1371/journal.pone.0106429)
Supplement: File S1 — includes the following: Data S1. Table presenting data underlying the results described in figure 1. Data S2. Table presenting data underlying the results described in figure 2. Data S3. Table presenting data underlying the results described in figure 3. Data S4. Table presenting data underlying the results described in figure 4. Data S5. Table presenting data underlying the results described in figure 5. Data S6. Table presenting data underlying the results described in figure 6. Data S7. Table presenting data underlying the results described in figure 7. Data S8. Table presenting data underlying the results described in figure 8. Data S9. Table presenting data underlying the results described in figure 9. Data S10. Table presenting data underlying the results described in figure 10. (DOC) [file pone.0106429.s001.doc]

**Data S1. Table presenting data underlying the results described in figure 1**

| Time on growth curve graph | | |  |  |  |  |  |  |  |
| --- | --- | --- | --- | --- | --- | --- | --- | --- | --- |
| Hours | Control | STDEV | ERROR | Hx 0,6 | STDEV | ERROR | Hx 1,5 | STDEV | ERROR |
| 0 | 0,0775 | 0,001915 | 0,000957 | 0,0785 | 0,001291 | 0,000645 | 0,07675 | 0,002062 | 0,001031 |
| 4,00 | 0,08125 | 0,0005 | 0,00025 | 0,0785 | 0,000577 | 0,000289 | 0,07875 | 0,0005 | 0,00025 |
| 8,00 | 0,08525 | 0,000957 | 0,000479 | 0,0825 | 0,000577 | 0,000289 | 0,0845 | 0,001291 | 0,000645 |
| 12,00 | 0,13575 | 0,002217 | 0,001109 | 0,1085 | 0,00238 | 0,00119 | 0,1155 | 0,001291 | 0,000645 |
| 16,00 | 0,22175 | 0,006185 | 0,003092 | 0,192 | 0,013736 | 0,006868 | 0,223 | 0,002944 | 0,001472 |
| 20,00 | 0,30925 | 0,006652 | 0,003326 | 0,28275 | 0,018464 | 0,009232 | 0,32525 | 0,00943 | 0,004715 |
| 24,0 | 0,41075 | 0,011206 | 0,005603 | 0,37675 | 0,01223 | 0,006115 | 0,4105 | 0,005508 | 0,002754 |
| 28,00 | 0,511 | 0,013491 | 0,006745 | 0,47025 | 0,009912 | 0,004956 | 0,4995 | 0,004203 | 0,002102 |
| 32,00 | 0,60625 | 0,00877 | 0,004385 | 0,55775 | 0,008302 | 0,004151 | 0,586 | 0,004761 | 0,00238 |
| 36,00 | 0,67475 | 0,008995 | 0,004498 | 0,63 | 0,005944 | 0,002972 | 0,65475 | 0,005058 | 0,002529 |
| 40,00 | 0,73075 | 0,007632 | 0,003816 | 0,6935 | 0,004933 | 0,002466 | 0,715 | 0,004619 | 0,002309 |
| 44,00 | 0,783 | 0,008124 | 0,004062 | 0,75275 | 0,005377 | 0,002689 | 0,77325 | 0,003202 | 0,001601 |
| 48,00 | 0,8255 | 0,007937 | 0,003969 | 0,8015 | 0,004203 | 0,002102 | 0,82 | 0,004761 | 0,00238 |
| 52,00 | 0,85675 | 0,008421 | 0,004211 | 0,837 | 0,003559 | 0,00178 | 0,8565 | 0,005686 | 0,002843 |
| 56,00 | 0,885 | 0,007572 | 0,003786 | 0,86975 | 0,0045 | 0,00225 | 0,889 | 0,006325 | 0,003162 |
| 60,00 | 0,8805 | 0,004655 | 0,002327 | 0,89075 | 0,005123 | 0,002562 | 0,90875 | 0,00789 | 0,003945 |
| 64,00 | 0,82475 | 0,006344 | 0,003172 | 0,88025 | 0,0105 | 0,00525 | 0,8825 | 0,021977 | 0,010989 |
| 68,00 | 0,77925 | 0,007136 | 0,003568 | 0,811 | 0,010328 | 0,005164 | 0,81675 | 0,021869 | 0,010934 |
| 72,00 | 0,74975 | 0,007042 | 0,003521 | 0,76075 | 0,007365 | 0,003683 | 0,7755 | 0,019841 | 0,009921 |
| 76,00 | 0,72825 | 0,008655 | 0,004328 | 0,73125 | 0,005679 | 0,002839 | 0,748 | 0,019782 | 0,009891 |
| 80,00 | 0,71125 | 0,008342 | 0,004171 | 0,7015 | 0,004509 | 0,002255 | 0,71575 | 0,0232 | 0,0116 |
| 84,00 | 0,6925 | 0,009983 | 0,004992 | 0,67575 | 0,006898 | 0,003449 | 0,68925 | 0,023796 | 0,011898 |
| 92,00 | 0,6595 | 0,011958 | 0,005979 | 0,634 | 0,006683 | 0,003342 | 0,65275 | 0,020549 | 0,010274 |
|  |  |  |  |  |  |  |  |  |  |
| Hours | Hx 5 | STDEV | ERROR | Hx 10 | STDEV | ERROR | Hx 20 | STDEV | ERROR |
| 0 | 0,07325 | 0,000957 | 0,000479 | 0,07475 | 0,0025 | 0,00125 | 0,067 | 0,000816 | 0,000408 |
| 4,00 | 0,079 | 0 | 0 | 0,07925 | 0,0025 | 0,00125 | 0,079 | 0 | 0 |
| 8,00 | 0,0795 | 0,000577 | 0,000289 | 0,07875 | 0,002217 | 0,001109 | 0,07825 | 0,0005 | 0,00025 |
| 12,00 | 0,0975 | 0,000577 | 0,000289 | 0,081 | 0,002708 | 0,001354 | 0,08025 | 0,0005 | 0,00025 |
| 16,00 | 0,16875 | 0,010308 | 0,005154 | 0,088 | 0,002708 | 0,001354 | 0,08525 | 0,0005 | 0,00025 |
| 20,00 | 0,25325 | 0,003775 | 0,001887 | 0,10875 | 0,002217 | 0,001109 | 0,101 | 0,000816 | 0,000408 |
| 24,0 | 0,35325 | 0,003862 | 0,001931 | 0,18925 | 0,011899 | 0,005949 | 0,16225 | 0,004425 | 0,002213 |
| 28,00 | 0,43075 | 0,000957 | 0,000479 | 0,2735 | 0,005447 | 0,002723 | 0,2115 | 0,006245 | 0,003122 |
| 32,00 | 0,51025 | 0,0015 | 0,00075 | 0,36325 | 0,004272 | 0,002136 | 0,28975 | 0,006652 | 0,003326 |
| 36,00 | 0,5825 | 0,002646 | 0,001323 | 0,43175 | 0,003202 | 0,001601 | 0,35075 | 0,003862 | 0,001931 |
| 40,00 | 0,64575 | 0,002872 | 0,001436 | 0,49975 | 0,0055 | 0,00275 | 0,41125 | 0,002986 | 0,001493 |
| 44,00 | 0,70875 | 0,0045 | 0,00225 | 0,5665 | 0,006403 | 0,003202 | 0,477 | 0,002828 | 0,001414 |
| 48,00 | 0,7595 | 0,005 | 0,0025 | 0,627 | 0,007394 | 0,003697 | 0,5335 | 0,002646 | 0,001323 |
| 52,00 | 0,79575 | 0,0045 | 0,00225 | 0,67675 | 0,007848 | 0,003924 | 0,5805 | 0,004203 | 0,002102 |
| 56,00 | 0,829 | 0,003559 | 0,00178 | 0,7205 | 0,007937 | 0,003969 | 0,626 | 0,003742 | 0,001871 |
| 60,00 | 0,86025 | 0,003775 | 0,001887 | 0,76225 | 0,010372 | 0,005186 | 0,66675 | 0,00263 | 0,001315 |
| 64,00 | 0,88525 | 0,0025 | 0,00125 | 0,79775 | 0,01021 | 0,005105 | 0,7005 | 0,003109 | 0,001555 |
| 68,00 | 0,876 | 0,009201 | 0,004601 | 0,8305 | 0,010599 | 0,005299 | 0,732 | 0,004 | 0,002 |
| 72,00 | 0,81225 | 0,014997 | 0,007499 | 0,855 | 0,011343 | 0,005672 | 0,7575 | 0,003873 | 0,001936 |
| 76,00 | 0,75875 | 0,006397 | 0,003198 | 0,8735 | 0,009 | 0,0045 | 0,77125 | 0,004113 | 0,002056 |
| 80,00 | 0,72775 | 0,00556 | 0,00278 | 0,85125 | 0,012842 | 0,006421 | 0,734 | 0,006633 | 0,003317 |
| 84,00 | 0,70275 | 0,004425 | 0,002213 | 0,784 | 0,014514 | 0,007257 | 0,67425 | 0,0025 | 0,00125 |
| 92,00 | 0,65125 | 0,006449 | 0,003224 | 0,70175 | 0,013937 | 0,006969 | 0,62575 | 0,00263 | 0,001315 |

**Data S2. Table presenting data underlying the results described in figure 2**

A)

| LB medium | % living cells |
| --- | --- |
| Control | 98,49094995 |
| Hx 10mM | 97,16448926 |
| Hx 20mM | 98,27077417 |

**B)**

| M9 medium | % living cells |
| --- | --- |
| Control | 87,3472712 |
| Hx 10mM | 88,8418339 |
| Hx 20mM | 56,4003207 |

**Data S3. Table presenting data underlying the results described in figure 3**

A)

| log cfu/ml | AVERAGE | STDEV | ERROR |
| --- | --- | --- | --- |
| Control | 58133333 | 6846897,1 | 2282299 |
| Hx 1,5mM | 51542857 | 13870706 | 3707102 |
| Hx 5mM | 52200000 | 4423080 | 1276833 |
| Hx 10mM | 43800000 | 6998181,6 | 2020201 |

**B)**

| *psyI* | AVERAGE | STDEV | ERROR |
| --- | --- | --- | --- |
| Control | 0,037747 | 0,003833 | 0,00271 |
| Hx 1,5mM | 0,018876 | 0,002532 | 0,001791 |
| Hx 5mM | 0,050719 | 0,038521 | 0,027238 |
| Hx 10mM | 0,055841 | 0,025005 | 0,017681 |

**Data S4. Table presenting data underlying the results described in figure 4**

**A)**

| *cfa1* | AVERAGE | STDEV | ERROR |
| --- | --- | --- | --- |
| Control | 0,87163413 | 0,061332 | 0,043369 |
| Hx 1,5mM | 0,83621605 | 0,052596 | 0,037191 |
| Hx 5mM | 1,0801549 | 0,300824 | 0,212715 |
| Hx 10mM | 1,75183458 | 0,23949 | 0,169345 |

**B)**

| *cmaB* | AVERAGE | STDEV | ERROR |
| --- | --- | --- | --- |
| Control | 0,0280959 | 0,0097052 | 0,0056033 |
| Hx 1,5mM | 0,0107399 | 0,016864 | 0,00840594 |
| Hx 5mM | 0,0207452 | 0,0226331 | 0,01153677 |
| Hx 10mM | 0,0156649 | 0,0087029 | 0,00502463 |

C)

| *cfl* | AVERAGE | STDEV | ERROR |
| --- | --- | --- | --- |
| Control | 0,36964767 | 0,114787 | 0,066272 |
| Hx 1,5mM | 0,51308246 | 0,001529 | 0,000883 |
| Hx 5mM | 0,50093552 | 0,341034 | 0,196896 |
| Hx 10mM | 0,72425774 | 0,203208 | 0,117322 |

**Data S5. Table presenting data underlying the results described in figure 5**

**A)**

| *hrpL* | AVERAGE | STDEV | ERROR |
| --- | --- | --- | --- |
| Control | 0,31773012 | 0,022808 | 0,013168 |
| Hx 1,5mM | 0,401997 | 0,170665 | 0,098533 |
| Hx 5mM | 0,3695008 | 0,11659 | 0,067313 |
| Hx 10mM | 0,81276536 | 0,26383 | 0,152322 |

B)

| *hrpA* | AVERAGE | STDEV | ERROR |
| --- | --- | --- | --- |
| Control | 0,18941576 | 0,004362 | 0,002518 |
| Hx 1,5mM | 0,1258067 | 0,034503 | 0,019921 |
| Hx 5mM | 0,23595242 | 0,05486 | 0,031674 |
| Hx 10mM | 0,64713077 | 0,24143 | 0,13939 |

C)

| *avrPtoB* | AVERAGE | STDEV | ERROR |
| --- | --- | --- | --- |
| Control | 0,45593316 | 0,173039 | 0,099904 |
| Hx 1,5mM | 0,64036826 | 0,073598 | 0,042492 |
| Hx 5mM | 0,57784004 | 0,281887 | 0,162748 |
| Hx 10mM | 1,02420424 | 0,404963 | 0,233805 |

D)

| *cmaX* | AVERAGE | STDEV | ERROR |
| --- | --- | --- | --- |
| Control | 1,04352495 | 0,316664 | 0,182826 |
| Hx 1,5mM | 1,09241338 | 0,586904 | 0,338849 |
| Hx 5mM | 0,90495928 | 0,113972 | 0,065802 |
| Hx 10mM | 1,24033603 | 0,338171 | 0,195243 |

**Data S6. Table presenting data underlying the results described in figure 6**

**A)**

|  | AVERAGE | STDEV | ERROR |
| --- | --- | --- | --- |
| Control inf | 88 | 12,6491106 | 4 |
| Hx inf | 37,3076923 | 17,3943699 | 4,82433019 |

B)

|  | 48h | 72h | STDEV 4 |  | ERROR |  |
| --- | --- | --- | --- | --- | --- | --- |
| Control inf | 11680000 | 3,36E+08 | 3077847 | 1,1E+08 | 3077851 | 28369131 |
| Hx inf | 4666667 | 8420000 | 870686,6 | 3553340 | 224810,3 | 1123665 |

**Data S7. Table presenting data underlying the results described in figure 7**

A)

| *psyI* | 48h | 72h | STDEV |  | ERROR |  |
| --- | --- | --- | --- | --- | --- | --- |
| Control inf | 3,960656 | 0,092143 | 5,753827 | 5,080587 | 0,332197374 | 0,254029 |
| Hx inf | 0,633783 | 0,004261 | 0,961615 | 0,845898 | 0,055518851 | 0,042295 |

B)

| *psrA* | 48h | 72h | STDEV |  | ERROR |  |
| --- | --- | --- | --- | --- | --- | --- |
| Control inf | 0,057073 | 0,001666 | 0,061462 | 0,070097 | 0,019436035 | 0,00701 |
| Hx inf | 0,001557 | 8,92E-07 | 0,00178 | 0,000584 | 0,000562992 | 0,000185 |

**Data S8. Table presenting data underlying the results described in figure 8**

A)

| *cfa1* | 48h | 72h | STDEV |  | ERROR |  |
| --- | --- | --- | --- | --- | --- | --- |
| Control inf | 103,5802 | 0,248038 | 74,83365 | 0,128235 | 43,20522651 | 0,074036 |
| Hx inf | 2,998079 | 0,066687 | 0,502513 | 0,128235 | 0,290125883 | 0,074036 |

B)

| *cmaB* | 48h | 72h | STDEV |  | ERROR |  |
| --- | --- | --- | --- | --- | --- | --- |
| Control inf | 0,02295 | 0,002263 | 0,018534 | 0,01833 | 0,005861018 | 0,002592 |
| Hx inf | 0,004409 | 0,031337 | 0,003746 | 0,013807 | 0,001184678 | 0,004366 |

C)

| *cfl* | 48h | 72h | STDEV |  | ERROR |  |
| --- | --- | --- | --- | --- | --- | --- |
| Control inf | 9,55194 | 4,145774 | 6,689102 | 0,922181 | 3,861954782 | 0,532422 |
| Hx inf | 2,765695 | 0,719519 | 4,473033 | 0,313251 | 2,58250669 | 0,180856 |

**Data S9. Table presenting data underlying the results described in figure 9**

**A)**

| *hrpL* | 48h | 72h | STDEV |  | ERROR |  |
| --- | --- | --- | --- | --- | --- | --- |
| Control inf | 0,036393 | 0,001374 | 0,032803 | 0,011361 | 0,010373309 | 0,003593 |
| Hx inf | 0,007881 | 0,034304 | 0,003307 | 0,016111 | 0,001045834 | 0,005095 |

B)

| *hrpA* | 48h | 72h | STDEV |  | ERROR |  |
| --- | --- | --- | --- | --- | --- | --- |
| Control inf | 0,490185 | 0,060427 | 0,20507 | 0,333602 | 0,064848968 | 0,047178 |
| Hx inf | 0,119273 | 0,51704 | 0,085796 | 0,258248 | 0,027131129 | 0,081665 |

C)

| *avrPtoB* | 48h | 72h | STDEV |  | ERROR |  |
| --- | --- | --- | --- | --- | --- | --- |
| Control inf | 0,247905 | 0,107279 | 0,319096 | 0,324298 | 0,045127045 | 0,045863 |
| Hx inf | 0,000266 | 0,001476 | 7,26E-05 | 0,000719 | 1,02684E-05 | 0,000102 |

D)

| *cmaX* | 48h | 72h | STDEV |  | ERROR |  |
| --- | --- | --- | --- | --- | --- | --- |
| Control inf | 10,63535 | 0,014245 | 11,8145 | 11,01168 | 1,181450372 | 0,34822 |
| Hx inf | 0,74243 | 0,016728 | 0,366456 | 0,470305 | 0,115883487 | 0,148724 |

**Data S10. Table presenting data underlying the results described in figure 10**

|  | 4dpi | 10 dpi | 20 dpi | 30dpi |
| --- | --- | --- | --- | --- |
| Control inf | 40511298 | 4449825 | 795122,8 | 688947,4 |
| Hx inf | 4374667 | 810963 | 7870,37 | 0 |
|  | STDEV |  |  |  |
| Control inf | 1,63E+08 | 2265515 | 1093962 | 461625 |
| Hx inf | 1031468 | 698779,3 | 22257,89 | 0 |
|  | ERROR |  |  |  |
| Control inf | 25789735 | 506584,5 | 244617,3 | 103222,5 |
| Hx inf | 230643,2 | 156251,8 | 4977,015 | 0 |
